# An *In Vitro* Evaluation of Leakage of Two Etch and Rinse and Two Self-Etch Adhesives after Thermocycling

**DOI:** 10.1155/2012/852841

**Published:** 2012-05-22

**Authors:** Sabine Geerts, Amandine Bolette, Laurence Seidel, Audrey Guéders

**Affiliations:** ^1^Division of Conservative and Adhesive Dentistry, Department of Dentistry, University of Liège, Quai G. Kurth, P.O. Box 45, 4020 Liège, Belgium; ^2^Department of Biostatistics, University of Liège, Quai G. Kurth, P.O. Box 45, 4020 Liège, Belgium

## Abstract

Our experiment evaluated the microleakage in resin composite restorations bonded to dental tissues with different adhesive systems. 40 class V cavities were prepared on the facial and lingual surfaces of each tooth with coronal margins in enamel and apical margins in cementum (root dentin). The teeth were restored with Z100 resin composite bonded with different adhesive systems: Scotchbond Multipurpose (*SBMP*), a 3-step Etch and Rinse adhesive, Adper Scotchbond 1 XT (*SB1*), a 2-step Etch and Rinse adhesive, AdheSE One (*ADSE-1*), a 1-step Self-Etch adhesive, and AdheSE (*ADSE*), a 2-step Self-Etch adhesive. Teeth were thermocycled and immersed in 50% silver nitrate solution. When both interfaces were considered, SBMP has exhibited significantly less microleakage than other adhesive systems (resp., for SB1, ADSE-1 and ADSE, *P* = 0.0007, *P* < 0.0001 and *P* < 0.0001). When enamel and dentin interfaces were evaluated separately, (1) for the Self-Etch adhesives, microleakage was found greater at enamel than at dentin interfaces (for ADSE, *P* = 0.024 and for ADSE-1, *P* < 0.0001); (2) for the Etch and Rinse adhesive systems, there was no significant difference between enamel and dentin interfaces; (3) SBMP was found significantly better than other adhesives both at enamel and dentin interfaces. In our experiment Etch and Rinse adhesives remain better than Self-Etch adhesives at enamel interface. In addition, there was no statistical difference between 1-step (ADSE-1) and 2-step (ADSE) Self-Etch adhesives.

## 1. Introduction

Currently, resin composites are more often used for direct posterior teeth restorations since many advances were made in adhesion and adhesives long-term performances. Adhesives are necessary to prevent leakage on resin composite restorations while dental composites are not able to bond to dental tissues. However, clinical microleakage remains the major cause for composite restorations failures implying postoperative sensibility, margin colorations, secondary decay, or pulpal inflammation [1–5]. Therefore, manufacturers have proposed many different adhesives involving different adhesion strategies. These adhesive systems were well described by Van Meerbeek et al. [6–8]: the Etch and Rinse (ER) adhesive systems (in three or two clinical steps), the Self-Etch (SE) adhesive systems (in two or one clinical step(s)), and the glass ionomer adhesives [6–9]. In their *in vitro* studies, several authors have reported different dental adhesive systems' bonding performance [10–24]. Therefore, results from thermocycling experiments have already pointed statistical differences between the ER adhesion strategy and the SE adhesion strategy [19, 25–27].

Therefore, the purpose of this study was to evaluate bonding performance of different dental adhesives after thermocycling: 2 ER adhesives (Scotchbond Multipurpose, *SBMP*—3M ESPE AG, Seefeld, Germany—a 3-step ER adhesive and Adper Scotchbond 1 × T, *SB1* 3M Espe AG, Seefeld, Germany—a 2-step ER adhesive) and 2 SE adhesives (AdheSE, *ADSE*—Ivoclar Vivadent AG, Schaan, Liechtenstein—a 2-step SE adhesive and AdheSE One, *ADSE-1*—Ivoclar Vivadent AG, Schaan, Liechtenstein—a 1-step adhesive) were evaluated according to the microleakage that was observed.

## 2. Materials and Methods

Twenty recently extracted human third molars were randomly selected for this experiment. The teeth were stored in a refrigerated saline solution for a maximum of 3 months as recommended by the ISO norms (ISO. Guidance on testing of adhesion to tooth structure. International Organization for Standardization. TR 11405,1-4, Geneva, Switzerland, 1994). All patients and an appropriate Ethical Committee have approved the collection of extracted teeth. Two cavities were drilled on the facial and the lingual sides of each tooth. All the cavities (*n* = 40) were rectangular, standardized for dimensions and shape (*h* × *w* × *l* = 2 mm × 2 mm × 3 mm) and were prepared with a cylindrical diamond bur (diameter = 0.9 mm) at the coronal-radicular junction: the margins were butt-jointed, half in the enamel and half in the root dentin. After that, the apices were fixed in an autopolymerizing resin (Paladur, Heraeus-Kulzer GmbH & Co. KG, Hanau, Germany).

The forty cavities were randomly assigned in 4 groups according to tested adhesive systems (Table 1):

- Scotchbond Multipurpose (SBMP) (3M ESPE AG, Dental products, Seefeld, Germany), a 3-step Etch and Rinse (ER) adhesive system;
- Adper Scotchbond 1 × T (SB1) (3M ESPE AG, Dental products, Seefeld, Germany), a 2-step Etch and Rinse (ER) adhesive system;
- AdhSE (ADSE) (Ivoclar Vivadent AG, Schaan, Liechtenstein), a 2-step Self-Etch (SE) adhesive system;
- AdhSE One (ADSE-1) (Ivoclar Vivadent AG, Schaan, Liechtenstein), a 1-step Self-Etch (SE) adhesive system.

**Figure 1:**
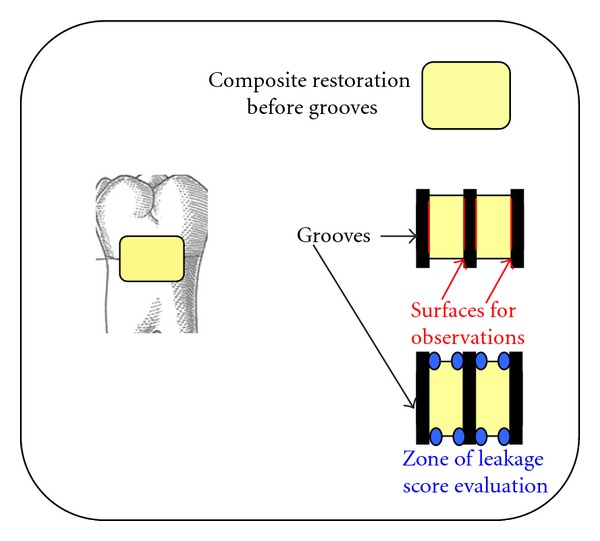
Diagram showing placement of the three grooves on each restoration to provide eight observation areas.

**Figure 2:**
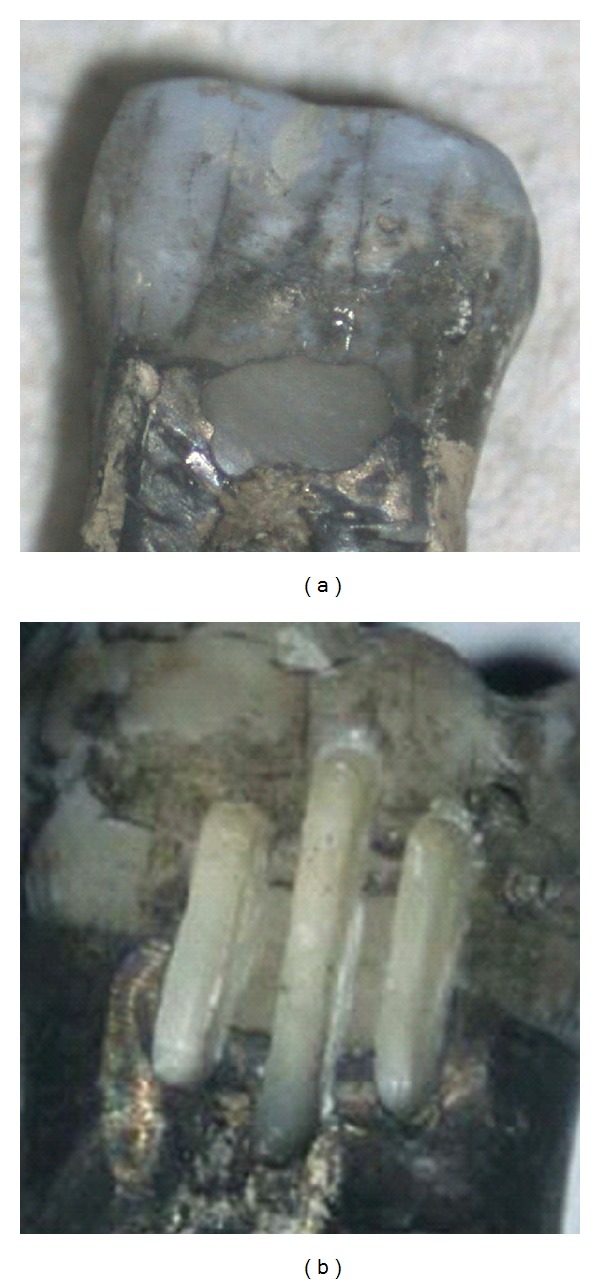
Pictures of the composite restoration before and after grooves' preparation.

All the tested adhesives were used according to the manufacturer's instructions. Immediately after bonding procedures, the cavities were filled with two oblique increments of a microhybrid composite (Z100, 3M ESPE AG, Dental products, Seefeld, Germany). The photopolymerization was carried out for all materials with a halogen lamp (XL 3000, 3M ESPE AG, Dental products, Seefeld, Germany). Composite restorations were polished by means of diamond drills and disks (Hawe Neos Dental, Bioggio, Switzerland). The polishing was carried out under a spray of water. After that, all the specimens were immersed in a saline solution for twelve weeks (in a refrigerator at 5°C). Thereafter, the teeth were thermocycled for 800 cycles (5°C–55°C) for 22 hours. After thermocycling, the teeth were immersed in a 50% silver nitrate solution (for 6 hours) and in a 25% vitamin C solution for 10 minutes (pH about 2) [25, 26]. After immersion, three grooves (3 mm depth, 1 mm width) were drilled with a diamond bur in each restoration to obtain four surfaces of observation. The interfaces that occurred between the teeth and the filling was described in our previous studies [25, 26]. Briefly, the cylindrical diamond drill (0.9 mm diameter) was placed perpendicular to the composite restoration. Three grooves 3 mm deep and 1 mm wide were cut on each restoration: one at the mesial margin, one at the distal margin, and one right in the middle of the filling (Figure 1). These preparations yielded four evaluating surfaces for each composite restoration (Figure 2), for a total of 160 viewing surfaces for all tested adhesives. Each surface allowed one observation in enamel and one in dentin (lecture areas), for a total of 320 observations (160 in enamel and 160 in dentin). 

Each section was examined by twofold magnification by means of an optic microscope (Carl Zeiss, SAS, Oberkochen, Germany). Each tooth was observed twice by the same operator (blinded test).

**Figure 3:**
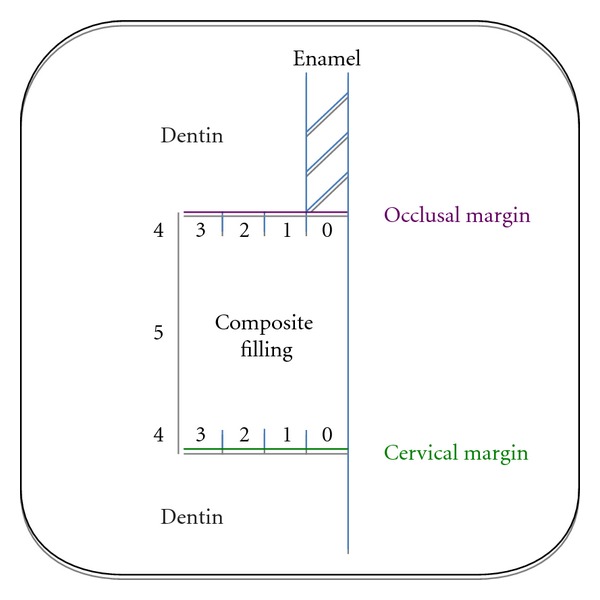
Diagram showing the 6-point evaluation scale for leakage.

Arbitrarily, the evaluation of leakage was made with a 6-point severity scale (Figure 3, Table 2) [25]. 

**Figure 4:**
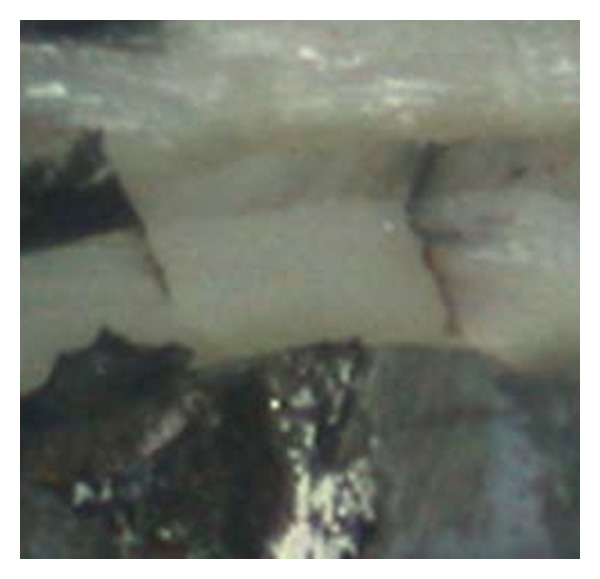
Picture showing a 2 score of microleakage (left) and 4 score of microleakage (right).

We have postulated that higher scores of microleakage (scores 3, 4, and 5) after thermocycling would be responsible for clinical failure of the bonding (Figure 4).

### 2.1. Statistical Analysis

Results are expressed as means ± standard deviations (± SDs). Microleakage scores were analyzed by means of generalized linear mixed models (GLMMs) assuming an ordinal logistic link function. Covariates in the model were (1) adhesive systems and (2) interface (enamel or dentin). The model also accounts for repeated measurements on the various teeth. All the results were considered to be significant at the 5% critical level (*P* < 0.05). Statistical calculations were made using the SAS 9.1 (version 8.2 for Windows) package.

## 3. Results

Microleakage mean score calculation for each tested adhesive system was analyzed by statistical model, which takes repeated evaluations into account (4 observations for each interface, enamel, or dentin). Therefore, all the observed scores of microleakage for each adhesive at enamel or at dentin interface are not displayed.

The mean scores of microleakage for all tested adhesive systems are shown in Table 3.

In our study, SBMP was significantly different from other adhesives: SBMP has shown a lower mean score of microleakage (0.30 ± 0.49) than other tested adhesives (*P* = 0.0007 for SB-1 and *P* < 0.0001 for the other tested adhesives).

Table 4 reports the statistical comparison between the mean scores of microleakage of the 4 tested adhesives.

As seen in Table 4, there was no statistical difference between SB1 and ADSE (*P* = 0.0799), neither between SB1 and ADSE-1 (*P* = 0.072) nor between ADSE and ADSE-1 (*P* = 0.96).

Table 5 shows the mean scores of microleakage for the 4 tested adhesives at enamel and dentin interfaces.

For ADSE and ADSE-1, the mean scores of microleakage were significantly lower at dentin than at enamel interfaces.

## 4. Discussion

For the past few years, composite has become current restorative material and today it often replaces amalgam restorations in posterior teeth [28–31]. Nevertheless, restorative composite is not able to bond to dental tissues. Therefore, the use of an adhesive system is always required. As result of numerous advances in adhesive technology and adhesion knowledge, there are many adhesive systems available on the market. To avoid confusing and incorrect uses of the adhesives, Professor Bart Van Meerbeek has proposed a classification according to different adhesion strategies and adhesives: the Etch and Rinse (ER) adhesive systems, the Self-Etch (SE) adhesive systems, and the glass ionomer adhesive systems [6, 7, 32]. The ER adhesives always involve the use of phosphoric acid, which permits demineralization of the dental tissues and, after rinsing, a complete elimination of the smear layer. Therefore, in the course of the ER adhesion strategy, the adhesive resin (bonding) is applied in a different clinical step: the demineralization and the hybridization of dental substrate appear consecutively. On the contrary, with the SE adhesives the demineralization and the impregnation of the adhesive into the enamel-dentin support appear simultaneously. The demineralization process results from the acidic monomers, which are components of the adhesive system. Therefore, the SE adhesive must not be rinsed. There are currently 4 different types of SE adhesives, which are indexed according to their pH value: the ultramild SE (pH about 2.5), the mild SE (pH about 2), the intermediary strong SE (pH about 1.5), and the strong SE (pH < 1) [6–8, 32]. On the enamel, for both ER and SE adhesive systems, bonding to the tissue is essentially micromechanical. On the dentin, for the ER adhesives, the mechanisms of adhesion are mainly micro-mechanical because the phosphoric acid is a very strong acid (pH about 0.5). Phosphoric acid completely dissolves the mineral and so, the collagen fibers are totally exposed after etching. For the SE adhesives, the adhesion to the dentin is both micro-mechanical and chemical [6–8]: the self-etch monomers are often less acidic than phosphoric acid and then some minerals remain attached to the collagen fibers, permitting chemical links between dental substrate and functional groups of the adhesive monomers.

Laboratory experiments have permitted comparison between different bonding materials and have pointed statistical differences between different adhesive systems [10–24]. Currently, a lot of studies and reviews agree about the best performances of the ER adhesive systems at the enamel and also at the dentin interface for some 3-step adhesives [11, 16, 19, 32–37]. Concerning dentin interface, several authors admit that some SE adhesives, in particular mild and ultra-mild, are able to create chemical bonds with hydroxyapatite crystals within the dentinal tissue [7, 8, 32, 36–40]. Nevertheless, some authors suggest that these mild and ultra-mild SE adhesives have poor adhesion capacity to the enamel tissue: so, they recommend the use of phosphoric acid on the enamel surface before applying the SE adhesive [32, 34, 37, 41–43].

Currently, *in vitro *microleakage [11, 19] and mechanical tests [16, 35] often show the superiority of the 3-step ER adhesives. For several authors, these adhesive systems are always the “gold standard” [6, 7, 32, 36, 37, 44], in particular the Optibond FL (*Kerr, European Union Representative, Scafati (SA), Italy*) [5, 7, 45] and/or the Scotchbond Multi-purpose Plus (SBMP) (*3 M Espe AG, Seefeld, Germany*) [5, 36, 44]. The results of our study are in agreement with the data from the literature: in our experiment, SBMP has shown the best results in terms of microleakage.

Nevertheless, for some authors, 2-step mild and ultra-mild SE adhesives can give comparable results than those obtained by some 2-step ER adhesives and also, by some 3-step ER adhesive systems [6–10, 32, 34, 36, 37]. In fact, our results have shown that ADSE (2-step SE) and SB1 (2-step ER) have statistically comparable mean scores of microleakage.

Concerning the 1-step SE adhesives, some *in vitro *studies have shown their poor performances [7, 34, 46]. Our results do not confirm this fact: there is no statistical difference between the mean scores of microleakage of ADSE (a 2-step SE) and its simplified clinical version, ADSE-1 (a 1-step SE). In addition, the mean scores of microleakage of these two mild SE adhesives (pH about 2) are lower at the dentin interface than at the enamel interface. These observations agree with data from the literature: ADSE and ADSE-1 are not efficient to create a sufficient micro-mechanical retention at the enamel surface [6, 7, 16, 34, 43]. Nevertheless, at the dentin surface, these mild SE adhesives are able to create a partial demineralization of this tissue to allow a micro-mechanical adhesion [6, 7]. In addition, some functional monomers of these SE adhesives might form chemical bonds with the calcium of the residual hydroxyapatite crystals linked to the collagen fibers [7, 32, 38–40]. The chemical bonds between ADSE functional monomers have not been clearly identified yet, but this adhesive has given good results in our study, like in the study of Bradna et al. [10].

## 5. Conclusion

In this study, confirming previous studies about marginal microleakage of the ER adhesive systems, SBMP, a 3-step ER adhesive, has significantly less microleakage comparing to other adhesive systems and can be considered like a reference adhesive. The parameters of this experiment (hydrolysis and thermocycling) have shown the good *in vitro *behaviour of SBMP. Therefore, we can expect that this ER adhesive will be clinically satisfying. In fact, this adhesive has been widely used for many years and their performances have seemed good. The 2-step ER adhesive that was tested in our study has shown a significantly greater mean score of microleakage than the tested 3-step ER adhesive system, but all the tested adhesives showed minimal leakage.

In the limits of our study, ADSE and ADSE-1 show poor microleakage, particularly on the dentin. Nevertheless, we suggest these mild SE adhesives can be used when the margins of the cavity are located on dentin and/or using phosphoric acid only on the enamel margin in order to optimize micro-mechanical interlocking.

## Figures and Tables

**Table 1:** The different tested adhesive systems and their components.

Adhesive systems		
Name of the adhesive	Type of adhesives	Components
SBMP	ER, 3 steps	Phosphoric acid (35%)
		Primer = HEMA, polyalkenoic acid copolymer, water
		Bonding = HEMA, Bis-GMA, amines, photoinitiator

SB1	ER, 2 steps	Phosphoric acid (35%)
		Adhesive (primer + bonding) = dimethacrylates, HEMA, polyalkenoic acid copolymer, silanized silicium, alcohol, water, photo-initiator

ADSE	SE, 2 steps	Primer = dimethacrylate, phosphonic acid acrylate, initiators, stabilizers
		Bonding = HEMA, dimethacrylate, silicon dioxide, Initiators, stabilizers

ADSE-1	SE, 1 step	Derivates of bis-acrylamide, water, alcohol, bis-methacrylamide dihydrogen phosphate, amino acid acrylamide, hydroxyl alkyl methacrylamide, alkyl sulfonic acid acrylamide, highly dispersed silicon dioxide, initiators, stabilizers, and potassium fluoride

In this table, the tested adhesives are displayed according to the adhesion's strategy and the type of adhesives: Etch and Rinse (ER) adhesive systems (SBMP and SB1) and Self-Etch adhesive systems (ADSE, ADSE-1). SBMP: Scotchbond Multipurpose Plus (3 M ESPE AG, Dental products, Seefeld, Germany). SB1: Adper Scotchbond 1 XT (3 M Espe AG, Dental products, Seefeld, Germany). ADSE = AdheSE (Ivoclar Vivadent AG, Schaan, Liechtenstein). ADSE-1: AdheSE One (Ivoclar Vivadent AG, Schaan, Liechtenstein).

**Table 2:** The 6-point severity scale to evaluate the microleakage.

Scores	Signification
Score = 0	No leakage
Score = 1	Leakage up to the enamel-dentin junction or a depth of 0.5 mm on the radicular wall
Score = 2	Leakage up to the maximum half of the lateral wall (leakage depth ≤1 mm)
Score = 3	Leakage over half of the lateral wall (1 mm < leakage depth < 2 mm)
Score = 4	Subtotal leakage on the whole of the lateral wall (leakage depth = 2 mm)
Score = 5	Total leakage partly or entirely on the pulpal wall of the cavity (leakage depth >2 mm)

**Table 3:** Mean microleakage scores of the different tested adhesive systems.

ER adhesive systems		SE adhesive systems	
	Mean microleakage scores (± SDs)		Mean microleakage scores (± SDs)
SBMP	0.30 ± 0.49	ADSE	0.88 ± 0.82
SB1	0.64 ± 0.66	ADSE-1	0.84 ± 0.72

In this table, the tested adhesives are displayed according to the adhesion's strategy and type of adhesives: Etch and Rinse (ER) adhesive systems (SBMP and SB1) and Self-Etch adhesive systems (ADSE, ADSE-1). SBMP. Scotchbond Multipurpose; SB1. Scotchbond 1 XT; ADSE. AdheSE; ADSE-1. AdheSE One.

**Table 4:** Statistical differences among the tested adhesives and level of significance (*P*).

	SBMP	SB1	ADSE	ADSE-1
SBMP	—			
SB1	0.0007	—		
ADSE	<0.0001	0.0799	—	
ADSE-1	<0.0001	0.072	0.96	—

SBMP. Scotchbond Multipurpose; SB1. Scotchbond 1 XT; ADSE, AdheSE; ADSE-1. AdheSE One.

**Table 5:** Mean microleakage scores of the tested adhesives at the enamel and dentin interfaces.

Strategy of adhesion	Adhesive systems	Mean microleakage scores (± SD)		*P*
		Enamel	Dentin	
ER	SBMP	0.30 ± 0.52	0.30 ± 0.46	0.86
	SB1	0.68 ± 0.73	0.60 ± 0.59	0.79
SE	ADSE	1.03 ± 0.70	0.73 ± 0.91	0.024
	ADSE-1	1.20 ± 0.65	1.48 ± 0.60	<0.0001

In this table, the tested adhesives are displayed according to the adhesion's strategy and type of adhesives: Etch and Rinse (ER) adhesive systems (SBMP and SB1) and Self-Etch adhesive systems (ADSE, ADSE-1).

SBMP: Scotchbond Multipurpose; SB1: Scotchbond 1 XT; ADSE: AdheSE; ADSE-1: AdheSE One.
